# Supplementary material for: Reduction in mitochondrial iron alleviates cardiac damage during injury
Source: EMBO Mol Med. 2016 Feb 19;8(3):247–67. doi: 10.15252/emmm.201505748 (PMC4772952; doi:10.15252/emmm.201505748)
Supplement: Supplementary file 1 — Appendix [file EMMM-8-247-s001.pdf]

Appendix

Reduction of Mitochondrial Iron Alleviates Cardiac Damage During Injury

Hsiang-Chun Chang [*, 1], Rongxue Wu [*, 1], Meng Shang[1], Tatsuya Sato[1], Chunlei Chen[1], Jason S. Shapiro[1], Ting Liu[1], Anita Thakur[1], Konrad T. Sawicki[1], Sathyamangla V. Naga Prasad [2], and Hossein Ardehali[1]

[1] Feinberg Cardiovascular Research Institute (FCVRI), Northwestern University Feinberg School of Medicine, Chicago, IL.

[2] Department of Molecular Cardiology, Lerner Research Institute, Cleveland Clinic Foundation, Cleveland, OH

[*]H.C. and R.W. contributed equally to this work

Table of Contents

Appendix Figure S1. Representative Western Blots from subcellular fractions.

Appendix Figure S2. Mitochondrial iron and TfR1 mRNA in response to BPD treatment in H9c2 cells.

Appendix Figure S3. ABCB8 levels in the hearts of cardiac-specific ABCB8 transgenic mice.

Appendix Figure S4. Cardiac function as assessed by fractional shortening in NTG and ABCB8 TG mice after I/R.

Appendix Figure S5. Cardiac function in mice treated with iron chelators at baseline and after I/R.

Appendix Figure S6. Deletion of ABCB8 in cardiac-specific ABCB8 knockout mice.

Appendix Figure S7. Effects of mitochondrial iron modulation on mitochondrial biogenesis or dynamics *in vitro*.

Appendix Figure S8. Effects of mitochondrial iron modulation on mitochondrial biogenesis and dynamics in wild-type mice with chelator treatment and ABCB8 TG mice.

Appendix Figure S9. Effects of mitochondrial iron modulation on mitochondrial biogenesis and dynamics after I/R *in vivo*.

Appendix Figure S10. Effects of mitochondrial iron modulation on the expression of NOS proteins, and genes involved in BH4 biosynthesis both at baseline and after I/R.

Appendix Table S1 Complete blood count and serum iron parameters in wild type mice with indicated chelator treatment

Appendix Table S2 Primer sequences for quantitative real-time PCR.

Appendix Table S3 Exact P-values.

Appendix Figure S1

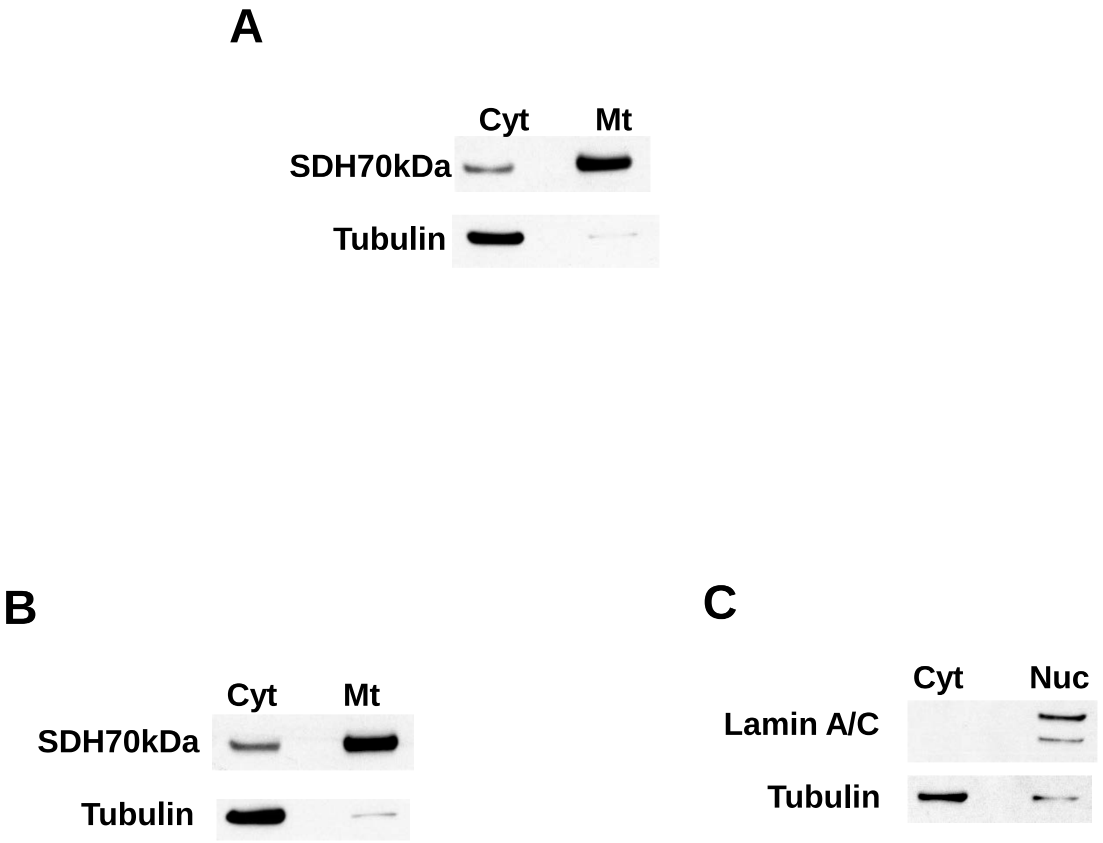

Appendix Figure S1. Representative Western Blots from subcellular fractions.

A. Presence of cytosolic marker (tubulin) and mitochondrial marker (SDH70kDa) in respective subcellular fractions from mouse heart. Equal amounts of protein from each fraction were loaded onto the gel. Cyt = cytosolic fraction. Mt = mitochondrial fraction.

B. Presence of cytosolic marker (tubulin) and mitochondrial marker (SDH70kDa) in respective subcellular fractions from human heart. Equal amounts of protein from each fraction were loaded onto the gel.

C. Presence of cytosolic marker (tubulin) and nuclear marker (Lamin A/C) in respective subcellular fractions from mouse heart. Cyt = cytosolic fraction. Nuc = nuclear fraction.

Appendix Figure S2

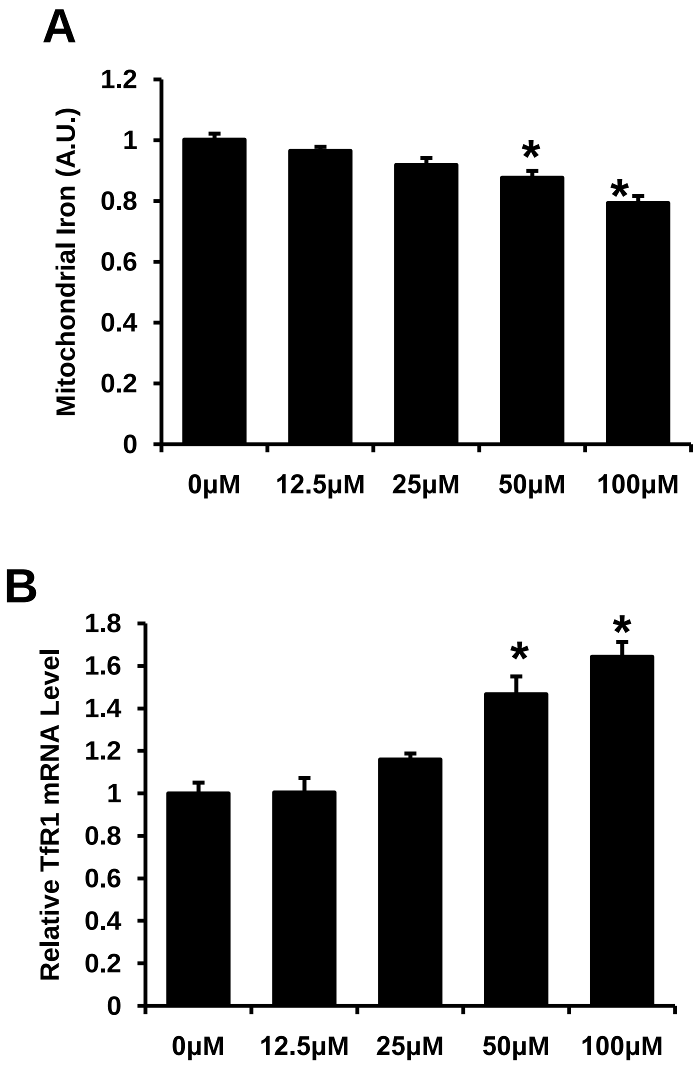

Appendix Figure S2. Mitochondrial iron and TfR1 mRNA in response to BPD treatment in H9c2 cells.

A. Mitochondrial iron levels in H9c2 cells treated with various doses of BPD. * $P<0.0001$ compared to no iron chelator treatment. N=6 independent samples in each group.

B. TfR1 expression, which increases in response to a decrease in cellular iron, in cells treated with various doses of BPD. * $P<0.0001$ compared to no treatment. N=6 independent samples in each group. All data are expressed as mean ± SEM. ANOVA and post-hoc Tukey test were performed. Exact P-values are reported in Appendix Table S3.

Appendix Figure S3

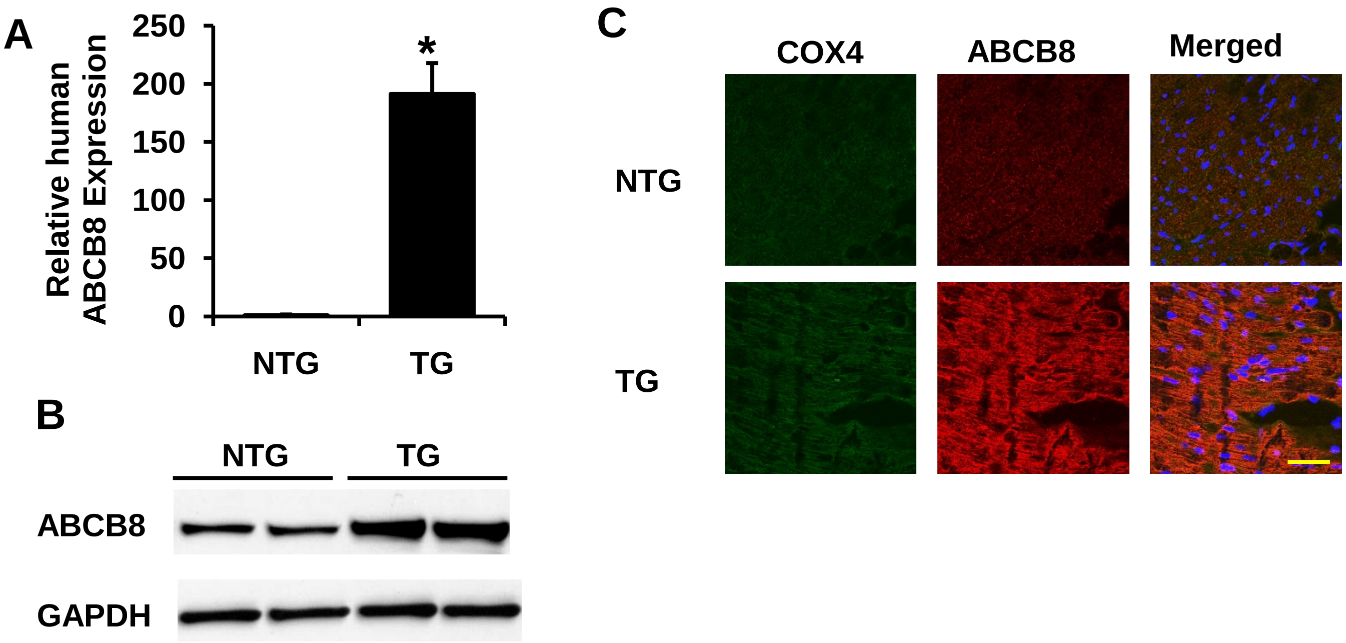

Appendix Figure S3. ABCB8 levels in the hearts of cardiac-specific ABCB8 transgenic mice.

A. mRNA levels of human ABCB8 in NTG and TG mouse hearts. N=6 mice for each group. * P= 0.0000295 for two-tailed unpaired t-test. Data are expressed as mean ± SEM.

B. ABCB8 protein levels in NTG and TG mouse hearts. N=2 mice in each group.

C. Representative confocal images from ABCB8 NTG and TG hearts staining for ABCB8 and COX4. Scale bar= 50µm.

Appendix Figure S4

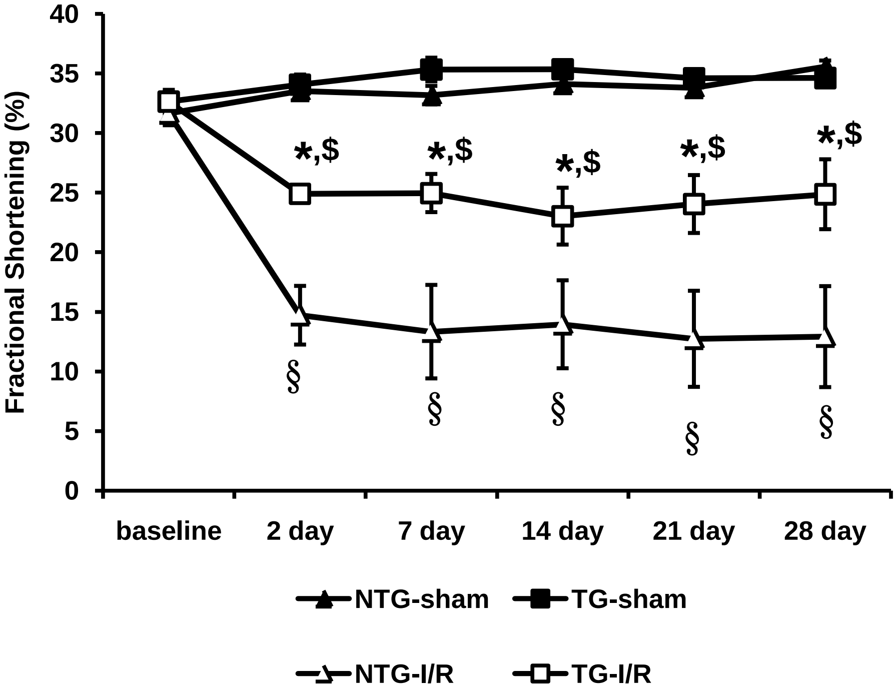

Appendix Figure S4. Cardiac function as assessed by fractional shortening in NTG and ABCB8 TG mice after I/R.

ANOVA and post-hoc Tukey test were performed. * P<0.05 TG-I/R compared with TG-sham at the same time point. $ P<0.01 TG-I/R compared with NTG-I/R at the same time point. § P<0.0001 NTG-I/R compared with NTG-sham at the same time point. Exact P-values are included in Appendix Table S3. N=5 mice for NTG-sham and NTG-I/R group and N=6 mice for TG-sham and TG-I/R group. Data are expressed as mean ± SEM.

Appendix Figure S5

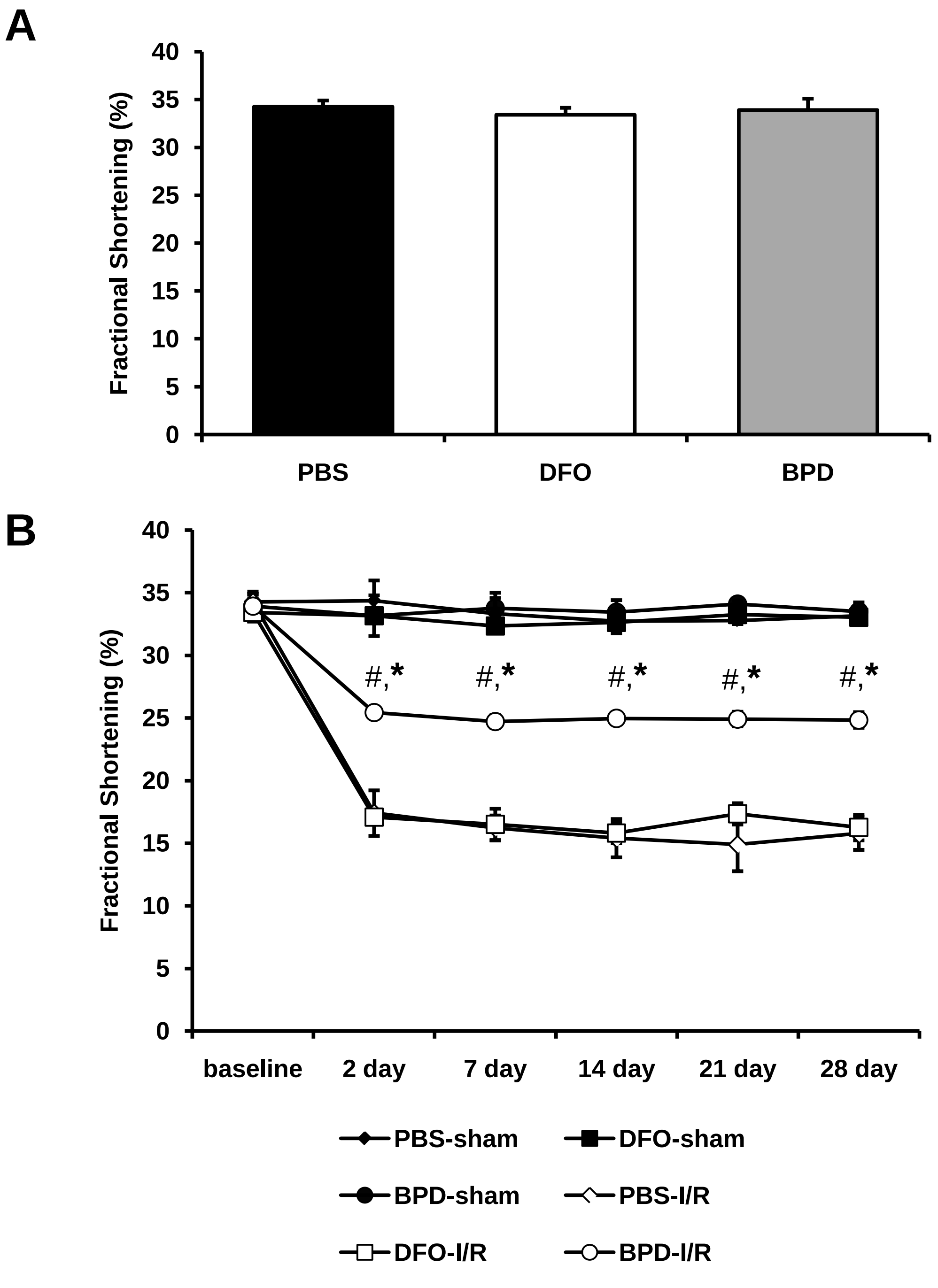

Appendix Figure S5. Cardiac function in mice treated with iron chelators at baseline and after I/R.

A. Fractional shortening in wild-type mice treated with indicated iron chelator for a week. ANOVA was performed. N=5 in each group.

B. Fractional shortening in wild-type mice pretreated with indicated iron chelator at different time points after I/R. ANOVA and post-hoc Tukey test were performed. Exact P-values are reported in Appendix Table S3. * P<0.0001 compared to PBS-I/R group at the same time point. # P<0.0001 compared to PBS-sham group at the same time point. N=5 mice PBS-sham and BPD-I/R, N=6 mice DFO-sham, BPD-sham, PBS-I/R, and DFO-I/R. All data are expressed as mean ± SEM.

Appendix Figure S6

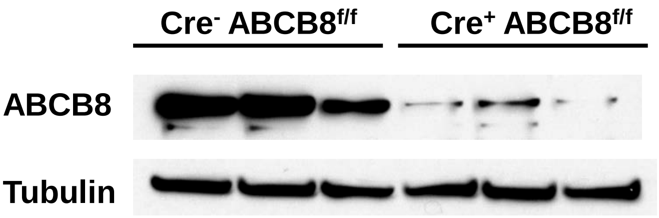

Appendix Figure S6. Deletion of ABCB8 in cardiac-specific ABCB8 knockout mice.
ABCB8 protein levels in Cre^{-} ABCB8$^{f/f}$ (referred to as WT in the manuscript) and Cre^{+} ABCB8$^{f/f}$ mice (referred to as KO in the manuscript) hearts after two weeks of tamoxifen treatment. N=3 mice in each group.

Appendix Figure S7

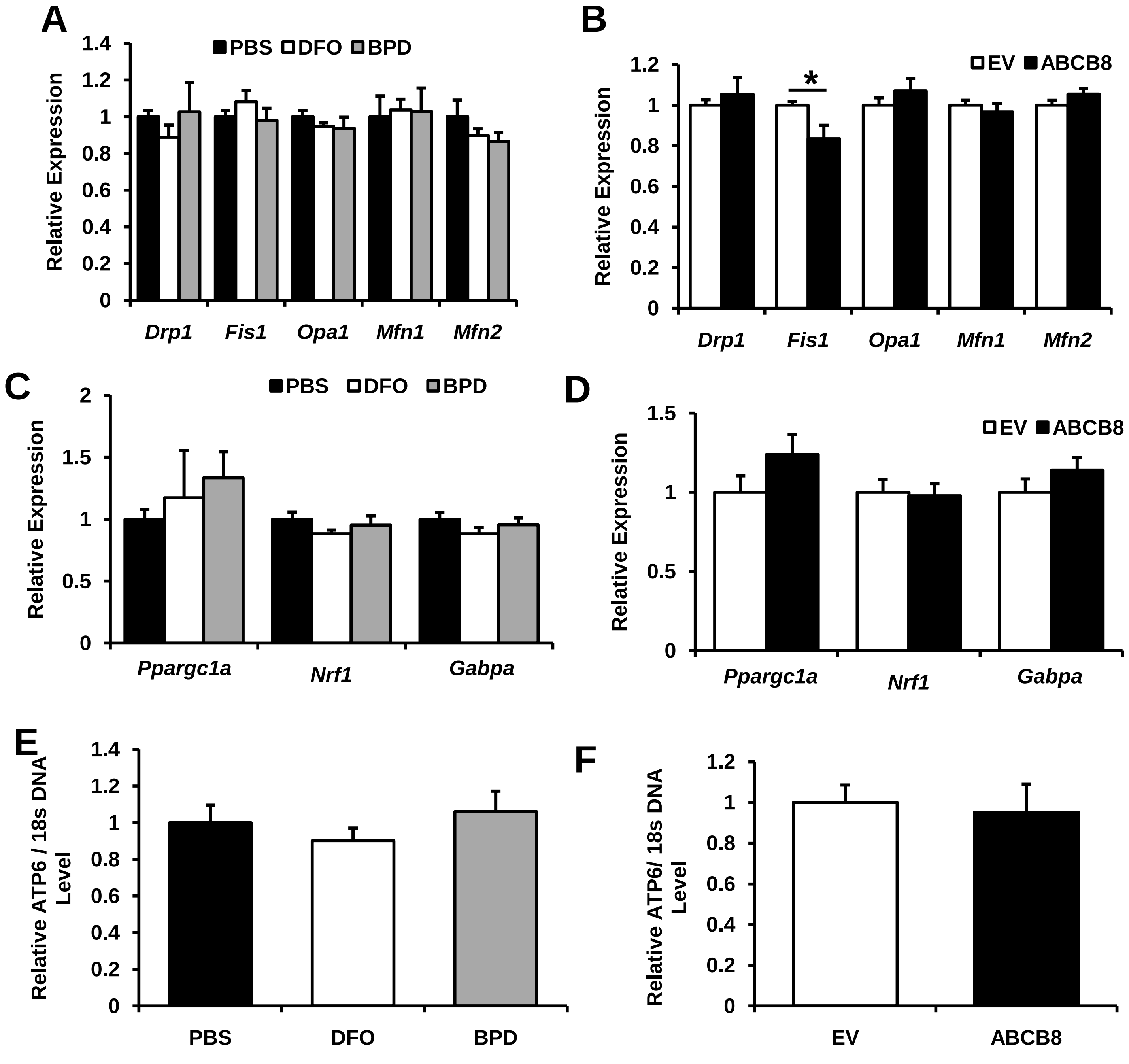

Appendix Figure S7. Effects of mitochondrial iron modulation on mitochondrial biogenesis or dynamics *in vitro*.

A. Expression of genes associated with mitochondrial dynamics in H9c2 cells treated with indicated iron chelator. ANOVA was performed. N=6 independent samples for PBS and N=5 independent samples for other groups.

B. Expression of genes associated with mitochondrial dynamics in H9c2 cells with ABCB8 overexpression. EV=empty vector. N=12 independent samples in each group. * P=0.026 with two-tailed unpaired T-test.

C. Expression of genes associated with mitochondrial biogenesis in H9c2 cells treated with indicated iron chelator. ANOVA was performed. N=6 independent samples for PBS and N=5 independent samples for other groups.

D. Expression of genes associated with mitochondrial biogenesis in H9c2 cells with ABCB8 overexpression. Two-tailed unpaired T-test was performed. N=12 independent samples in each group.

E. Mitochondrial DNA content in H9c2 cells treated with indicated iron chelator. ANOVA was performed. N=6 independent samples for all groups.

F. Mitochondrial DNA content in H9c2 cells with ABCB8 overexpression. Two-tailed unpaired T-test was performed. N=6 independent samples in each group. All data are expressed as mean ± SEM.

Appendix Figure S8

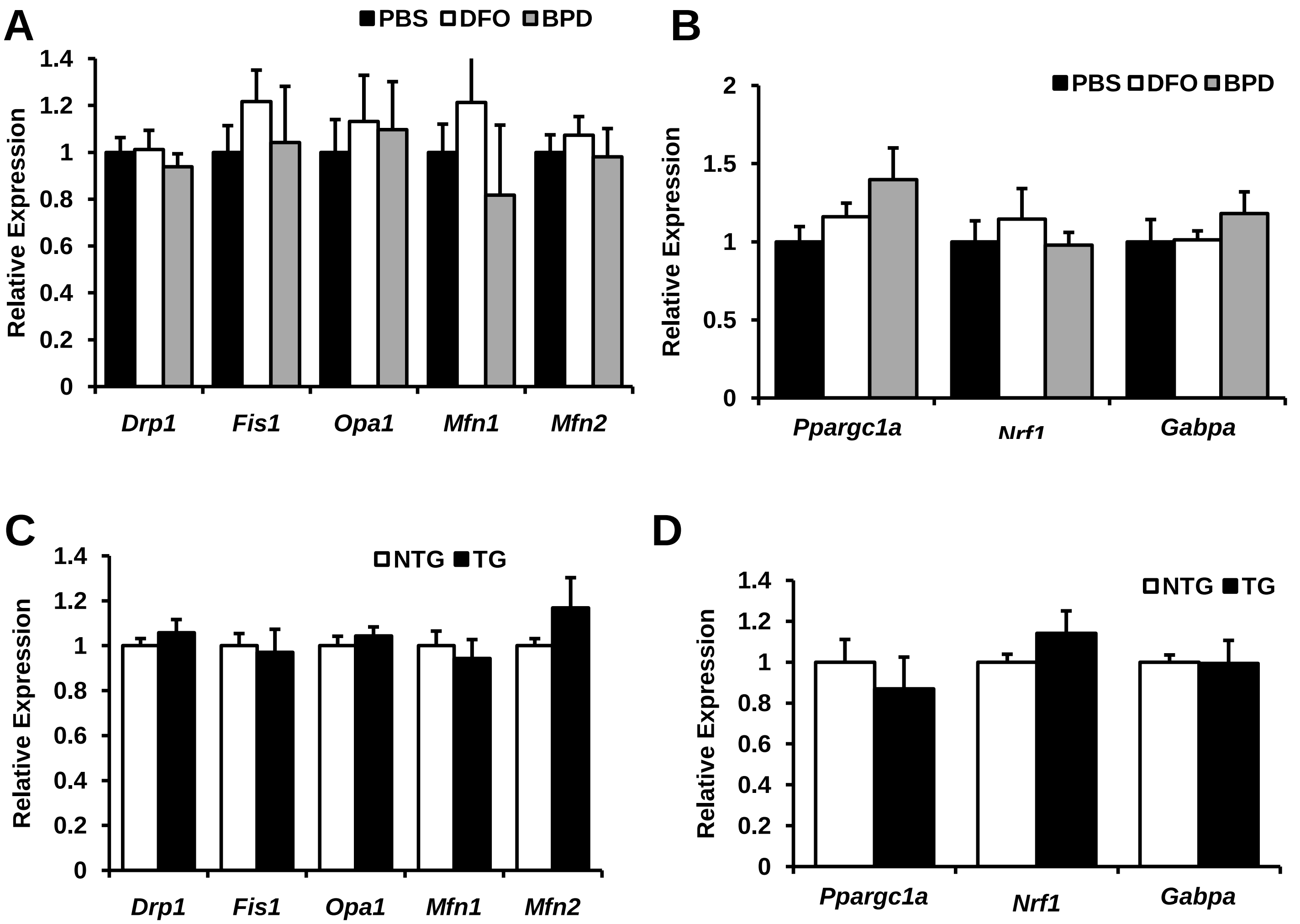

Appendix Figure S8. Effects of mitochondrial iron modulation on mitochondrial biogenesis and dynamics in wild-type mice with chelator treatment and ABCB8 TG mice.

A. Expression of genes associated with mitochondrial dynamics in wild-type mice treated with indicated iron chelator. ANOVA was performed. N=5 mice in each group.

B. Expression of genes associated with mitochondrial biogenesis in wild-type mice treated with indicated iron chelator. ANOVA was performed. N=5 mice in each group.

C. Expression of genes associated with mitochondrial dynamics in ABCB8 TG mice. Two-tailed unpaired T-test was performed. N=6 mice in each group.

D. Expression of genes associated with mitochondrial biogenesis in ABCB8 TG mice. Two-tailed unpaired T-test was performed. N=6 mice in each group. All data are expressed as mean ± SEM.

Appendix Figure S9

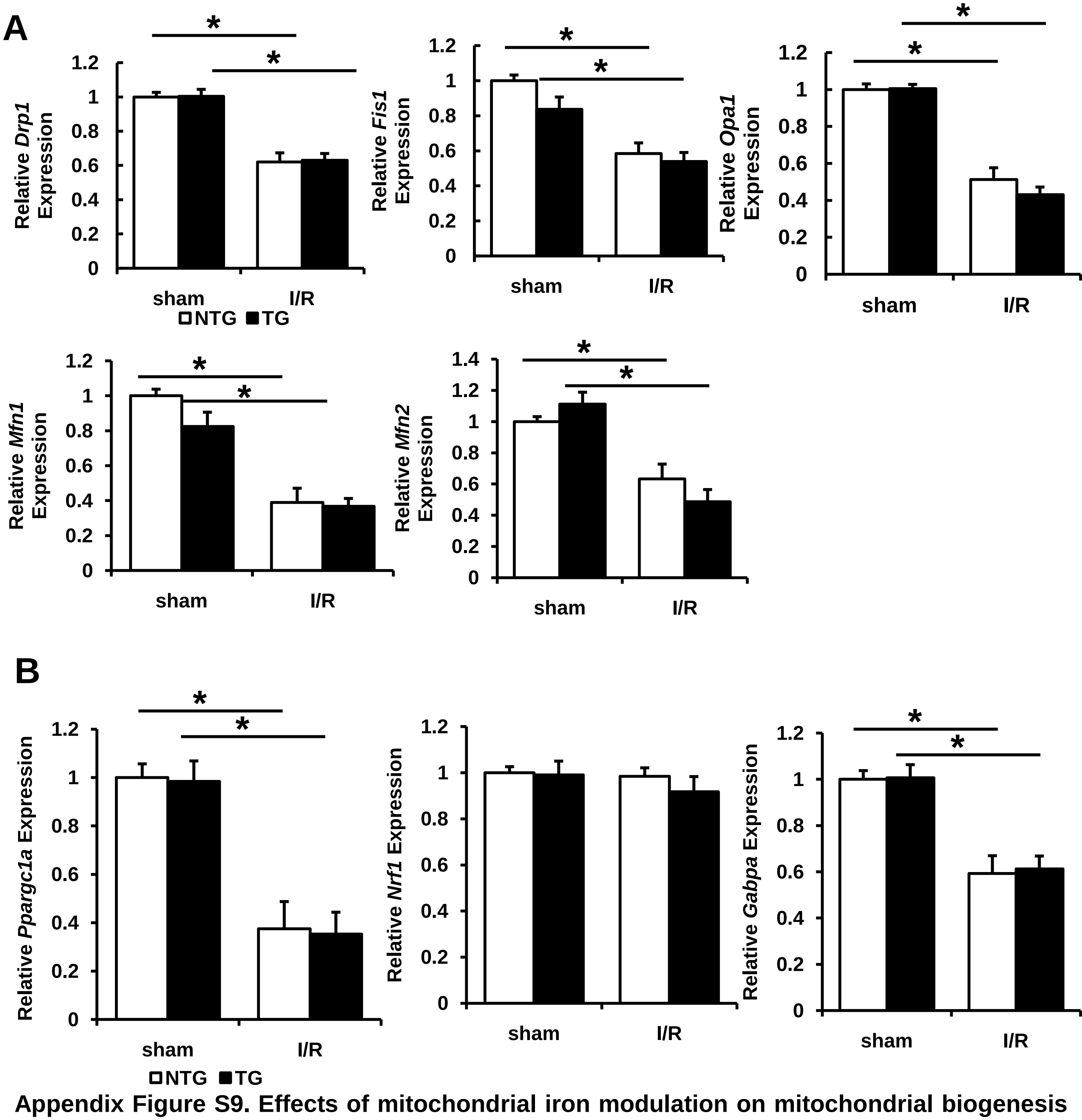

Appendix Figure S9. Effects of mitochondrial iron modulation on mitochondrial biogenesis and dynamics after I/R *in vivo*.

A. Expression of genes associated with mitochondrial dynamics in ABCB8 TG mice subjected to indicated procedure. N=12 mice for NTG-sham and TG-sham, and N=10 mice for NTG-I/R and TG-I/R. * P<0.05. ANOVA and post-hoc Tukey test were performed. Exact P-values are reported in Appendix Table S3.

B. Expression of genes associated with mitochondrial biogenesis in ABCB8 TG mice subjected to indicated procedure. N=12 mice for NTG-sham and TG-sham, and N=10 mice for NTG-I/R and TG-I/R. * P< 0.05. ANOVA and post-hoc Tukey test were performed. Exact P-values are reported in Appendix Table S3. All data are expressed as mean ± SEM.

Appendix Figure S10

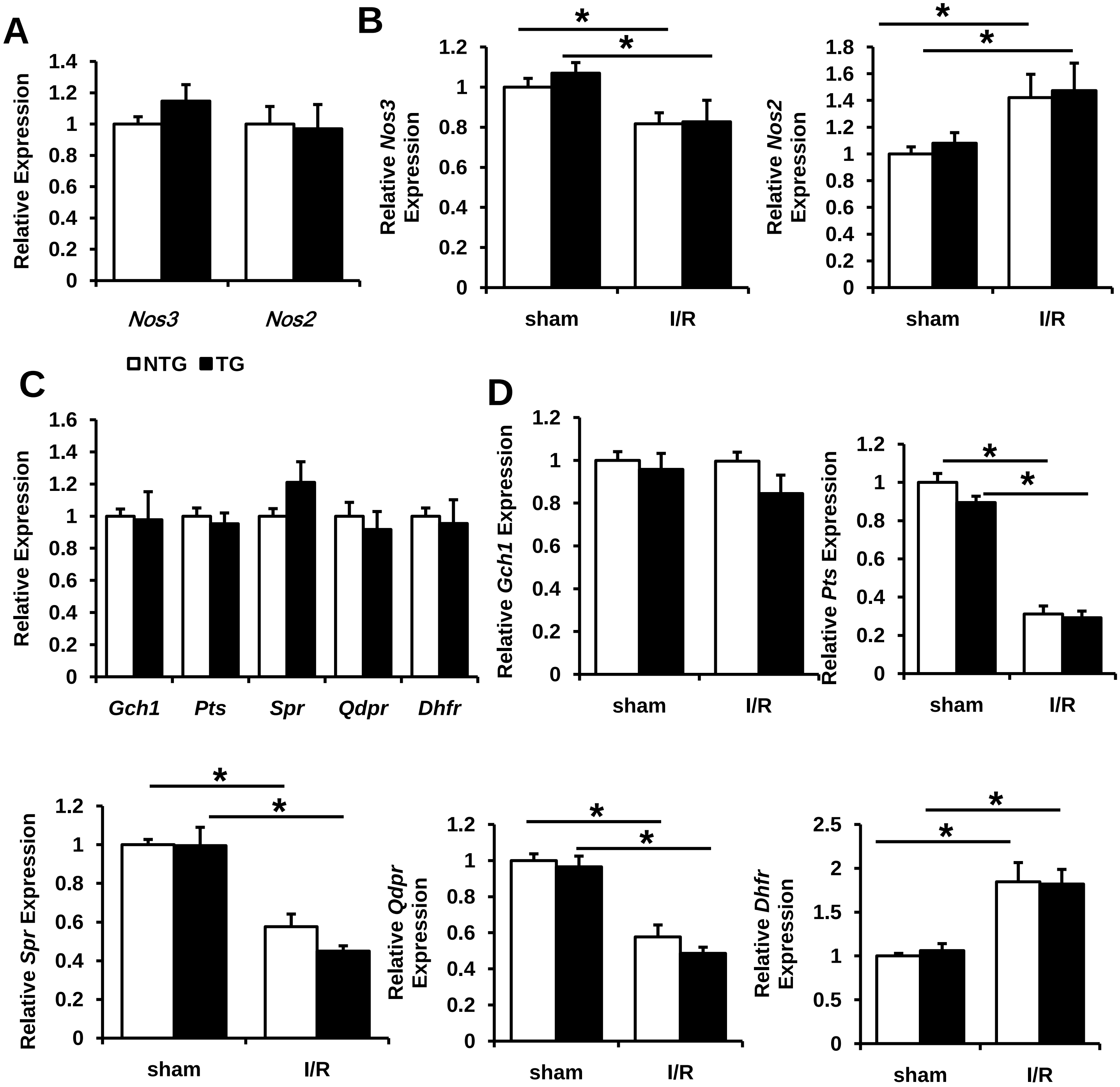

Appendix Figure S10. Effects of mitochondrial iron modulation on the expression of NOS proteins, and genes involved in BH4 biosynthesis both at baseline and after I/R.

A. Expression of NOS in ABCB8 TG mice at baseline. Two-tailed unpaired T-test was performed. N=6 mice in each group.

B. Expression of NOS in ABCB8 TG mice 2 day after I/R. N=12 mice for NTG-sham and TG-sham, N=10 mice for NTG-I/R and TG-I/R. ANOVA and post-hoc Tukey test were performed. * $P < 0.05$. Exact P-values are reported in Appendix Table S3.

C. Expression of genes involved in BH4 biosynthesis in ABCB8 TG mice at baseline. Two-tailed unpaired T-test was performed. N=6 mice in each group.

D. Expression of genes involved in BH4 biosynthesis in ABCB8 TG 2 day after I/R. N=12 mice for NTG-sham and TG-sham, N=10 mice for NTG-I/R and TG-I/R. ANOVA and post-hoc Tukey test were performed. * $P < 0.05$. Exact P-values are reported in Appendix Table S3. All data are expressed as mean ± SEM.

Appendix Table S1 Complete blood count and serum iron parameters in wild-type mice with indicated chelator treatment (n=5 mice in each group).

	PBS	DFO	BPD
RBC (10^6/µL)	9.02 ± 0.34	9.77 ± 0.43	10.7 ± 0.62
Hemoglobin (g/dL)	11.66 ± 0.46	12.96 ± 0.53	13.12 ± 0.30
Hematocrit (%)	42.42 ± 1.55	47.02 ± 2.25	47.53 ± 1.84
MCV (fL)	47.06 ± 0.54	48.1 ± 0.31	47.02 ± 0.36
MCH (pg)	12.92 ± 0.13	13.28 ± 0.12	12.38 ± 0.53
MCHC (g/dL)	27.52 ± 0.59	27.62 ± 0.32	26.34 ± 1.21
RDW (%)	16.2 ± 0.2	17 ± 0.32	17 ± 0.41
WBC (10^3/µL)	5.64 ± 0.97	6.62 ± 0.85	5.70 ± 0.40
Platelet (10^3/µL)	629.6 ± 128.61	711.6 ± 139.86	1007 ± 236.72
Serum Iron (µg/dL)	77.62 ± 5.38	53.37 ± 6.63 *	44.40 ± 2.24 *
Unsaturated Iron Binding Capacity (µg/dL)	200.96 ± 12.56	227.85 ± 7.71	247.02 ± 4.93 *
Total Iron Binding Capacity (µg/dL)	278.58 ± 10.63	281.21 ± 12.76	291.42 ± 4.63
Iron Saturation (%)	28.07 ± 2.24	18.66 ± 1.68 *	15.25 ± 0.81 *

ANOVA with post-hoc Tukey test was performed. * P=0.01 serum iron DFO vs. PBS. * P=0.002 serum iron BPD vs. PBS. * P=0.005 iron saturation DFO vs. PBS. * P=0.0004 iron saturation BPD vs. PBS.

Appendix Table S2. Primer sequences for quantitative real-time PCR.

Mouse Primers

Gene Name	Forward Primer	Reverse Primer
18s	AGTCCCTGCCCTTTGTACACA	CGATCCGAGGGCCTCACTA
Actb	CCGTGAAAAGATGACCCAGAT	GTACATGGCTGGGGTGTTG
Hprt	CTGGAAAGAATGTCTTGATTGTTG	TGCATTGTTTTACCAGTGTCAA
B2m	TGGTCTTTCTGGTGCTTGTC	CAGTTCAGTATGTTCGGCTTC
Tfr1	GCATTGCGGACTGTAGAGG	GCTTGATCCATCATTCTCAGC
Nppa	GGGTAGGATTGACAGGATTGG	CCTCCTTGGCTGTTATCTTC
Nppb	ATCCGTCAGTCGTTTGGG	CAGAGTCAGAAACTGGAGTC
Myh7	TGTTTCCTTACTTGCTACCC	GGATTCTCAAACGTGTCTAGTG
Sod1	TGCAGGGAACCATCCACTTC	ATGCTGGCCTTCAGTTAATCC
Sod2	GCTCTAATCAGGACCCATTG	TACTGAAGGTAGTAAGCGTG
Hmox1	AGGCTTTAAGCTGGTGATGG	CTTCCAGGGCCGTGTAGATA
Hmox2	TGGCACCAGAAAAGGAAAAC	CTTCCTTGGTCCCTTCCTTC
Cat	GTGCGCTGTAGATGTGAAAC	CCTCGTTCAACACCTTTGTG
Fis1	AATATGCCTGGTGCCTGGTT	GCTGTTCCTCTTTGCTCCCT
Drp1	AGGTTGCCCGTGACAAATGA	TCAGCAAAGTCGGGGTGTTT
Opa1	GTGACTATAAGTGGATTGTGCCTG	AACTGGCAAGGTCTTCTGAGC
Mfn1	ACTCAGTAAACGTGGCAGCA	TCCTCCGTGACCTCCTTGAT
Mfn2	GGCCTACATCCAAGAGAGCG	GCTGATACCCCTGACCTTGG
Nos3	TGGATGAGTATGATGTGGTGTCC	CTAGGGGAGCTGTTGTACGG

Nos2	TCTTTGACGCTCGGAACTGT	GTCATGTTTGCCGTCACTCC
Ppargc1a	GACTGGCGTCATTCGGG	ATCAAGTTCAGAAAGGTCAAGTT
Nrf1	AGCATGATCCTGGAAGACCTCG	TGGGATAAATGCCCGAAGCTG
Gabpa	CGCCTTGGCATCCCCTAT	CCACCCAATGCAGGACTTG
Gch1	AGCGCCTCACCAAACAGATT	TTTCTGCACGCCTCGCATTA
Pts	TGGTTATGAATTTGACCGACCTCAA	CACATCCAGGTCCAGGTTCTTG
Spr	TACAAAGGCTGGGGTCTGTACT	CAACTGCTGCATGTCATTGTCC
Qdpr	GCTCCTGGACACCCTTAGAG	TTCCTGAGTTTGGCCGTTTG
Dhfr	AATGACCACAACCTCTTCAGTG	CTCCTCGTGGTGGTTCTTTG

Rat Primers

Gene Name	Forward Primer	Reverse Primer
18s	AGTCCCTGCCCTTTGTACACA	CGATCCGAGGGCCTCACTA
Actb	GGCTCCTAGCACCATGAAGA	CAGTGAGGCCAGGATAGAGC
Hprt	CCCTCAGTCCCAGCGTCGTG	CGAGCAAGTCTTTCAGTCCTGTCC
B2m	CCGTGATCTTTCTGGTGCTTG	GAGACACGTAGCAGTTGAGGA
Tfr1	ATGGATCAAGCCAGATCAGC	AATGGCTCCCCTCCAAACA
Fis1	GCTGTCCTGAGATCATCCTCG	GCGTATTCAAACTGCGTGCT
Drp1	TCGTAAAAGGTTGCCCGTGA	TCAGCAAAGTCGGGGTGTTT
Opa1	TGACAAAGGCATCCACCACA	AACAACCCGTGGCAGATGAT
Mfn1	AAGGCCATCACTGCGATCTT	GCGAGCTTGTTTCTGTAGCC
Mfn2	CAAGGTCAGGGGAATCAGCG	TCACGGTGCTCTTCCCATTG
Ppargc1a	CCGCACACATCGCAATTC	CGGCTGTAGGGTGACCTTGA

Nrf1	AGCCATTGTTCTCTGCATCTCA	TGCGCCAAACACCTTAAAGA
Gabpa	TCCAGCATCAGTGCCTTCTG	GCTGCCTTTGCGCTACTGTT
Gch1	GCCATGCAGTTCTTCACCAAG	AATGGGACCAGGTGATGCTC
Pts	GGTGACAATTCATGGAGAGATCG	TCAAGGGGCTTCATAATGGC
Spr	TGGCTGTTGAGGAACCCAGT	AGTCTGCTCCTCAACTCTGG
Qdpr	GCTCCTGGACACCCTTAGAG	TTCCTGAGTTTGGCCGTTTG
Dhfr	ACATGGTCTGGGTAGTCGGAG	ACAAAGAGTCTGAGGTGGCCT

Appendix Table S3 Exact P-values

Figure 4A

2 day	P-value
TG-I/R vs. NTG-I/R	1.75E-04
TG-sham vs. TG-I/R	7.39E-04
NTG-sham vs. NTG-I/R	2.01E-07

7 day	P-value
TG-I/R vs. NTG-I/R	4.68E-04
TG-sham vs. TG-I/R	1.66E-02
NTG-sham vs. NTG-I/R	2.40E-06

14 day	P-value
TG-I/R vs. NTG-I/R	5.42E-03
TG-sham vs. TG-I/R	6.23E-03
NTG-sham vs. NTG-I/R	5.99E-06

21 day	P-value
TG-I/R vs. NTG-I/R	7.10E-04
TG-sham vs. TG-I/R	2.21E-02
NTG-sham vs. NTG-I/R	3.18E-06

28 day	P-value
TG-I/R vs. NTG-I/R	1.28E-03
TG-sham vs. TG-I/R	6.65E-06
NTG-sham vs. NTG-I/R	0.06925

**Appendix Figure S2A**	P-value
50µM vs. no treatment	9.25E-04
100 µM vs. no treament	9.70E-08

**Appendix Figure S2B**	P-value
50µM vs. no treatment	2.22E-04
100 µM vs. no treament	1.92E-06

Figure 6E

2 day	P-value
BPD-I/R vs. PBS-I/R	5.29E-05
BPD-I/R vs. PBS-sham	3.40E-04

7 day	P-value
BPD-I/R vs. PBS-I/R	3.81E-06
BPD-I/R vs. PBS-sham	6.81E-05

14 day	P-value
BPD-I/R vs. PBS-I/R	7.56E-07
BPD-I/R vs. PBS-sham	5.19E-04

21 day	P-value
BPD-I/R vs. PBS-I/R	9.29E-06
BPD-I/R vs. PBS-sham	4.29E-03

28 day	P-value
BPD-I/R vs. PBS-I/R	1.20E-06
BPD-I/R vs. PBS-sham	1.08E-04

Appendix Figure S4

2 Day	P-value
TG I/R vs TG sham	3.84E-04
TG I/R vs. NTG I/R	4.27E-04
NTG I/R vs. NTG sham	2.66E-07
7 Day	P-value
TG I/R vs TG sham	1.03E-02
TG I/R vs. NTG I/R	9.16E-04
NTG I/R vs. NTG sham	4.51E-06
14 Day	P-value
TG I/R vs TG sham	2.41E-03
TG I/R vs. NTG I/R	6.58E-03
NTG I/R vs. NTG sham	3.49E-06
21 Day	P-value
TG I/R vs TG sham	1.31E-02
TG I/R vs. NTG I/R	7.57E-04
NTG I/R vs. NTG sham	2.23E-06
28 Day	P-value
TG I/R vs TG sham	4.13E-02
TG I/R vs. NTG I/R	1.21E-03
NTG I/R vs. NTG sham	3.52E-06

Appendix Figure S5B

2 Day	P-value
BPD I/R vs. PBS I/R	1.70E-04
BPD I/R vs. PBS sham	7.10E-05
7 Day	P-value
BPD I/R vs. PBS I/R	5.13E-06
BPD I/R vs. PBS sham	8.63E-06
14 Day	P-value
BPD I/R vs. PBS I/R	5.56E-07
BPD I/R vs. PBS sham	3.97E-05
21 Day	P-value
BPD I/R vs. PBS I/R	3.64E-06
BPD I/R vs. PBS sham	3.14E-04
28 Day	P-value
BPD I/R vs. PBS I/R	6.84E-07
BPD I/R vs. PBS sham	6.57E-06

Appendix Figure S9A

Drp1	P-value
NTG sham vs. NTG I/R	5.30E-08
TG sham vs. TG I/R	1.84E-07
Fis1	P-value
NTG sham vs. NTG I/R	6.69E-06
TG sham vs. TG I/R	2.21E-03
Opa1	P-value
NTG sham vs. NTG I/R	0
TG sham vs. TG I/R	1.08E-07
Mfn1	P-value
NTG sham vs. NTG I/R	1.65E-07
TG sham vs. TG I/R	9.03E-05
Mfn2	P-value
NTG sham vs. NTG I/R	0.00171
TG sham vs. TG I/R	3.81E-07

Appendix Figure S9B

Ppargc1a	P-value
NTG sham vs. NTG I/R	2.28E-05
TG sham vs. TG I/R	2.44E-05
Gabpa	P-value
NTG sham vs. NTG I/R	3.57E-05
TG sham vs. TG I/R	7.62E-05

Appendix Figure S10B

Nos3	P-value
NTG sham vs. NTG I/R	0.0397
TG sham vs. TG I/R	0.01095
Nos2	P-value
NTG sham vs. NTG I/R	0.00947
TG sham vs. TG I/R	0.02739

Appendix Figure S10D

Pts	P-value
NTG sham vs. NTG I/R	1.19E-07
TG sham vs. TG I/R	2.61E-10
Spr	P-value
NTG sham vs. NTG I/R	1.68E-04
TG sham vs. TG I/R	2.97E-06
Qdpr	P-value
NTG sham vs. NTG I/R	3.18E-06
TG sham vs. TG I/R	3.42E-07
Dhfr	P-value
NTG sham vs. NTG I/R	1.96E-05
TG sham vs. TG I/R	4.72E-04
