# Supplementary material for: Reduction in mitochondrial iron alleviates cardiac damage during injury
Source: EMBO Mol Med. 2016 Feb 19;8(3):247–67. doi: 10.15252/emmm.201505748 (PMC4772952; doi:10.15252/emmm.201505748)
Supplement: Supplementary file 2 — Source Data for Appendix [file EMMM-8-247-s002.zip › Source_data_ appendix_figures/Source_data_for_ appendix_figure_S1.pptx]

## Slide 1
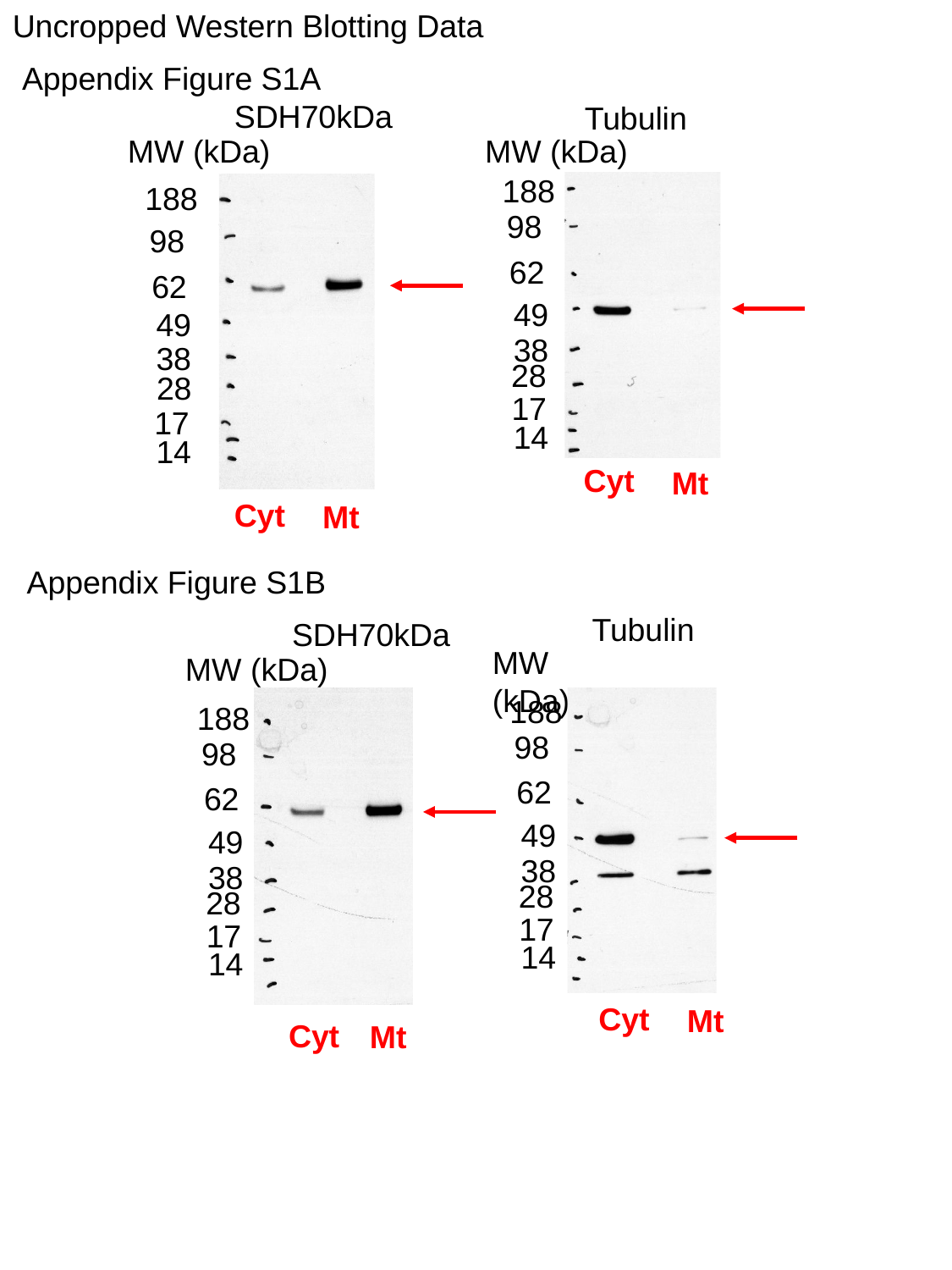

Uncropped Western Blotting Data
Appendix Figure S1A
SDH70kDa
Tubulin
MW (kDa)
MW (kDa)
188
188
98
98
62
62
49
49
38
38
28
28
17
17
14
14
Cyt
Mt
Cyt
Mt
Appendix Figure S1B
Tubulin
SDH70kDa
MW (kDa)
MW (kDa)
188
188
98
98
62
62
49
49
38
38
28
28
17
17
14
14
Cyt
Mt
Cyt
Mt

## Slide 2
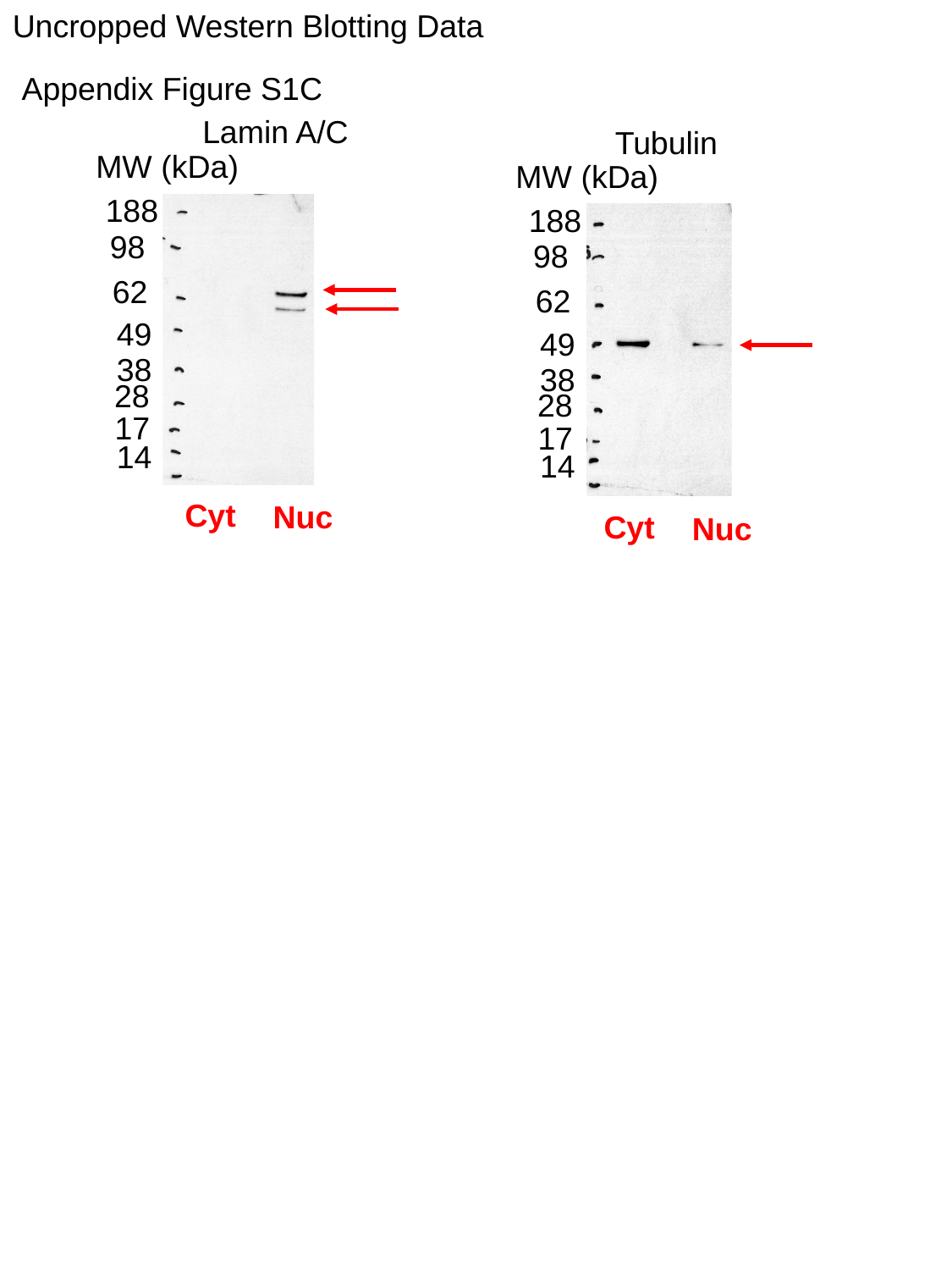

Uncropped Western Blotting Data
Appendix Figure S1C
Lamin A/C
Tubulin
MW (kDa)
MW (kDa)
188
188
98
98
62
62
49
49
38
38
28
28
17
17
14
14
Cyt
Nuc
Cyt
Nuc
