# Supplementary material for: Reduction in mitochondrial iron alleviates cardiac damage during injury
Source: EMBO Mol Med. 2016 Feb 19;8(3):247–67. doi: 10.15252/emmm.201505748 (PMC4772952; doi:10.15252/emmm.201505748)
Supplement: Supplementary file 2 — Source Data for Appendix [file EMMM-8-247-s002.zip › Source_data_ appendix_figures/Source_data_for_ appendix_figure_S6.pptx]

## Slide 1
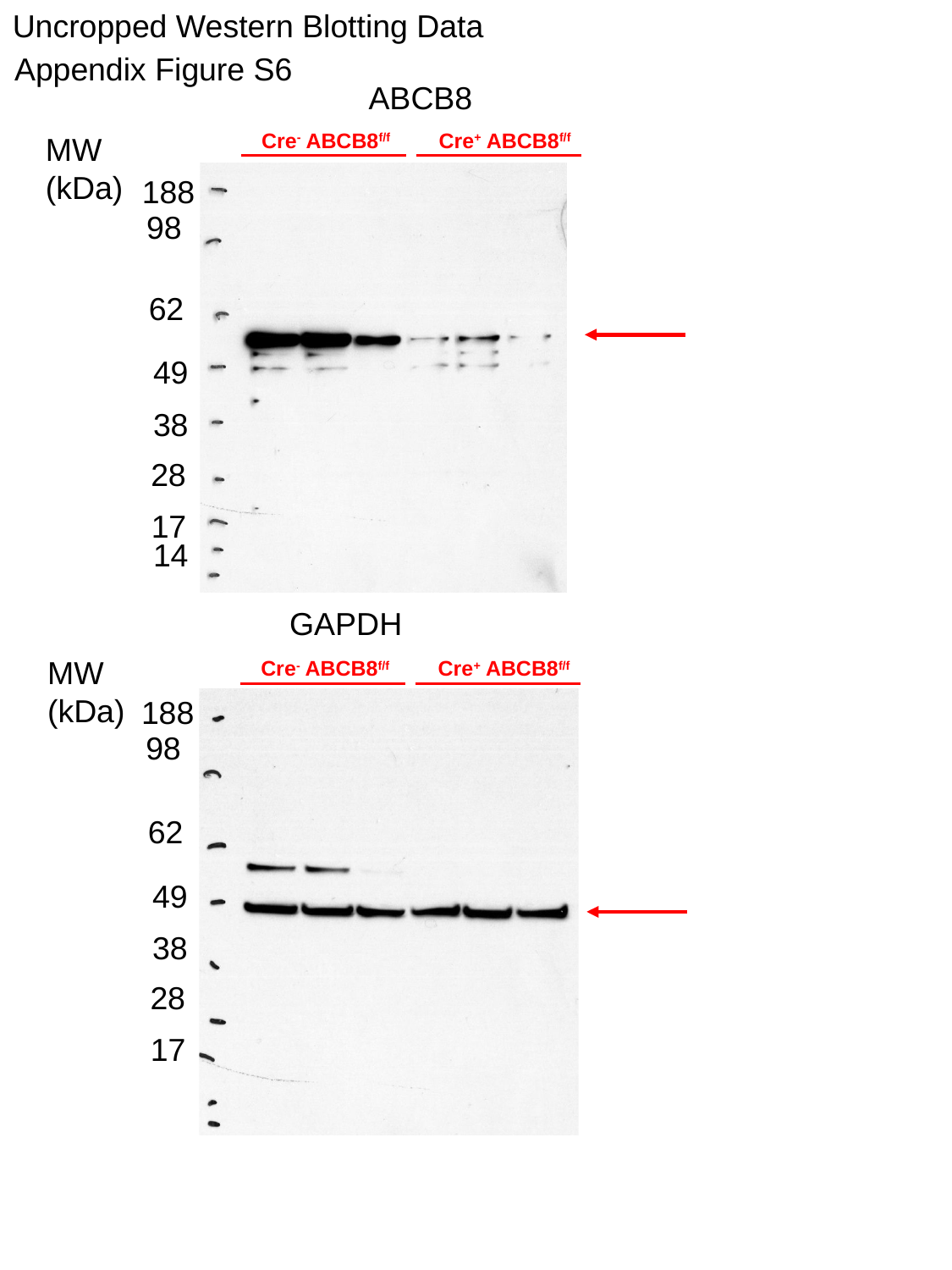

Uncropped Western Blotting Data
Appendix Figure S6
ABCB8
Cre- ABCB8f/f
Cre+ ABCB8f/f
MW (kDa)
188
98
62
49
38
28
17
14
GAPDH
MW (kDa)
Cre- ABCB8f/f
Cre+ ABCB8f/f
188
98
62
49
38
28
17
