# Supplementary figures and images for: Reduction in mitochondrial iron alleviates cardiac damage during injury
Source: EMBO Mol Med. 2016 Feb 19;8(3):247–67. doi: 10.15252/emmm.201505748 (PMC4772952; doi:10.15252/emmm.201505748)

## Slide 1
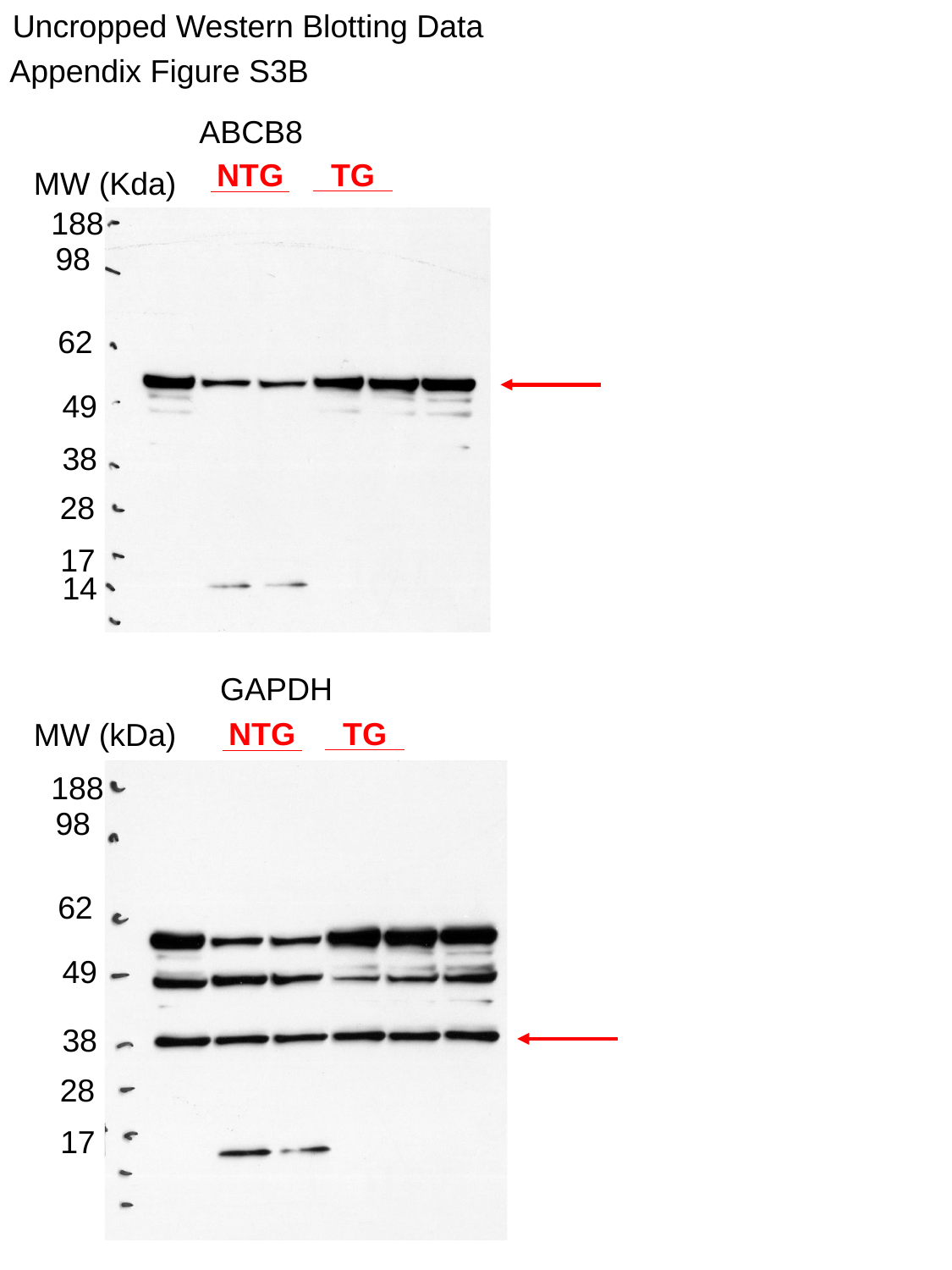

Uncropped Western Blotting Data
Appendix Figure S3B
ABCB8
NTG
TG
MW (Kda)
188
98
62
49
38
28
17
14
GAPDH
NTG
TG
MW (kDa)
188
98
62
49
38
28
17

Supplement: Supplementary file 2 — Source Data for Appendix [file EMMM-8-247-s002.zip › Source_data_ appendix_figures/Source_data_for_appendix_figure_S3.pptx]

## Slide 1
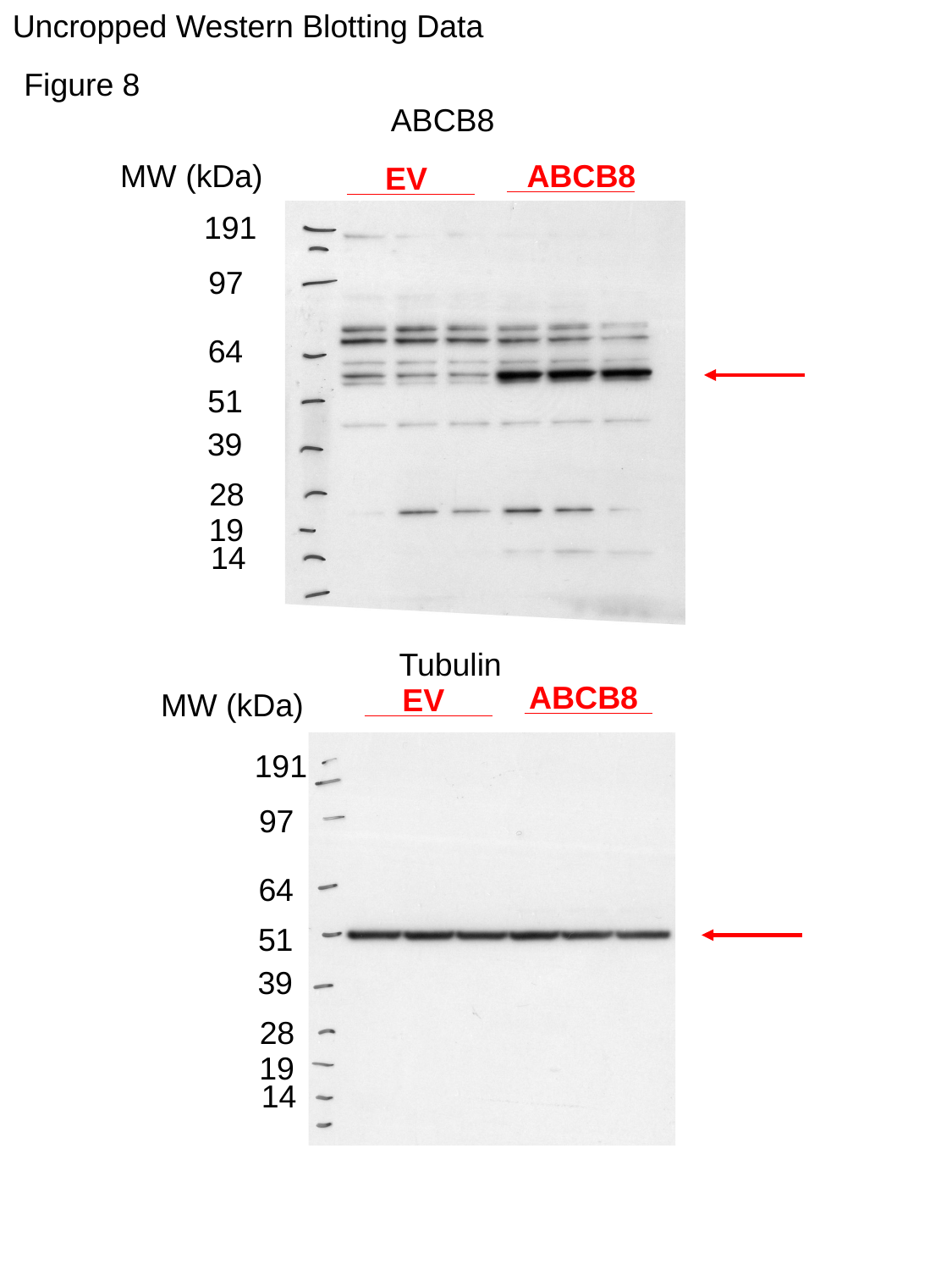

Uncropped Western Blotting Data
Figure 8
ABCB8
MW (kDa)
ABCB8
EV
191
97
64
51
39
28
19
14
Tubulin
ABCB8
EV
MW (kDa)
191
97
64
51
39
28
19
14

Supplement: Supplementary file 4 — Source Data for Figure 8 [file EMMM-8-247-s003.pptx]
